# Antigenic Pressure on H3N2 Influenza Virus Drift Strains Imposes Constraints on Binding to Sialylated Receptors but Not Phosphorylated Glycans

**DOI:** 10.1128/JVI.01178-19

**Published:** 2019-10-29

**Authors:** Lauren Byrd-Leotis, Chao Gao, Nan Jia, Akul Y. Mehta, Jessica Trost, Sandra F. Cummings, Jamie Heimburg-Molinaro, Richard D. Cummings, David A. Steinhauer

**Affiliations:** a Department of Microbiology and Immunology, Emory University School of Medicine, Atlanta, Georgia, USA; b Beth Israel Deaconess Medical Center, Department of Surgery and Harvard Medical School Center for Glycoscience, Harvard Medical School, Boston, Massachusetts, USA; c Centers for Excellence in Influenza Research and Surveillance, Emory-UGA CEIRS, Atlanta, Georgia, USA; St. Jude Children's Research Hospital

**Keywords:** phosphorylated glycans, receptor binding, sialylated glycans, antigenic drift, influenza

## Abstract

H3N2 strains of influenza A virus emerged in humans in 1968 and have continued to circulate, evolving in response to human immune pressure. During this process of “antigenic drift,” viruses have progressively lost the ability to agglutinate erythrocytes of various species and to replicate efficiently under the established conditions for amplifying clinical isolates and generating vaccine candidates. We have determined the glycome profiles of chicken and guinea pig erythrocytes to gain insights into reduced agglutination properties displayed by drifted strains and show that both chicken and guinea pig erythrocytes contain complex sialylated N-glycans but that they differ with respect to the extent of branching, core fucosylation, and the abundance of poly-N-acetyllactosamine (PL) [-3Galβ1-4GlcNAcβ1-]_n_ structures. We also examined binding of the H3N2 viruses using three different glycan microarrays: the synthetic Consortium for Functional Glycomics array; the defined N-glycan array designed to reveal contributions to binding based on sialic acid linkage type, branched structures, and core modifications; and the human lung shotgun glycan microarray. The results demonstrate that H3N2 viruses have progressively lost their capacity to bind nearly all canonical sialylated receptors other than a selection of biantennary structures and PL structures with or without sialic acid. Significantly, all viruses displayed robust binding to nonsialylated high-mannose phosphorylated glycans, even as the recognition of sialylated structures is decreased through antigenic drift.

**IMPORTANCE** Influenza subtype H3N2 viruses have circulated in humans for over 50 years, continuing to cause annual epidemics. Such viruses have undergone antigenic drift in response to immune pressure, reducing the protective effects of preexisting immunity to previously circulating H3N2 strains. The changes in hemagglutinin (HA) affiliated with drift have implications for the receptor binding properties of these viruses, affecting virus replication in the culture systems commonly used to generate and amplify vaccine strains. Therefore, the antigenic properties of the vaccines may not directly reflect those of the circulating strains from which they were derived, compromising vaccine efficacy. In order to reproducibly provide effective vaccines, it will be critical to understand the interrelationships between binding, antigenicity, and replication properties in different growth substrates.

## Abstract (precis)

Influenza subtype H3N2 viruses have circulated in humans for over 50 years, continuing to cause annual epidemics. Such viruses have undergone antigenic drift in response to immune pressure, reducing the protective effects of preexisting immunity to previously circulating H3N2 strains. The changes in hemagglutinin (HA) affiliated with drift have implications for the receptor binding properties of these viruses, affecting virus replication in the culture systems commonly used to generate and amplify vaccine strains. Therefore, the antigenic properties of the vaccines may not directly reflect those of the circulating strains from which they were derived, compromising vaccine efficacy. In order to reproducibly provide effective vaccines, it will be critical to understand the interrelationships between binding, antigenicity, and replication properties in different growth substrates.

## INTRODUCTION

Circulating influenza A viruses (IAV) present a substantial public health burden every year which has been exacerbated in years such as 2017 to 2018 when vaccine efficacy was impacted by the circulation of “antigenically drifted” H3N2 virus strains. H3N2 viruses first emerged in humans with the 1968 pandemic. When the structure of H3 hemagglutinin (HA) was determined and the mutations that had accumulated due to human immunity were mapped in the 1980s, five antigenic sites on the globular head domain were identified (1–3). The viruses have continued to evolve and now have mutations at over 100 amino acid positions (4). With the exception of a handful of conserved residues in the receptor binding site (RBS), the mutations cover virtually the entire surface of the HA head domains. A number of mutations are at positions around the edge of the pocket where sialic acid makes contacts and, as such, have the potential to influence HA interactions with receptors. The link between antigenicity and altered receptor binding properties can have far-reaching consequences for diagnostics and vaccine production (5). The general receptor binding characteristics of seasonal strains have been well established. IAVs bind to glycans terminating in sialic acid, and numerous groups have demonstrated the species specificity of sialic acid recognition: avian viruses are more specific for α2,3-linked sialic acid (α2,3-Sia), and human and swine viruses prefer α2,6-linked sialic acid (α2,6-Sia) (6–11).

The discrimination for linkage specificity is not an all-or-none phenomenon, and, in fact, nuclear magnetic resonance (NMR) studies of binding of avian or human HAs to monosialylated receptor analogs of each linkage type showed only a 2-fold-to-3-fold increase in binding affinities for the preferred linkage type (12, 13). Though the 1968 human viruses preferred α2,6-linked receptors, they maintained a limited capacity to recognize particular α2,3-linked glycans. This recognition gradually decreased as H3 strains circulated and adapted to human hosts until 2001, when H3 HAs had lost α2,3-Sia recognition altogether (4, 14). The reduction in preference for the avian-type receptor corresponded to a reduction in agglutination of chicken erythrocytes commonly used in hemagglutination and hemagglutination inhibition (HI) assays (15, 16) and poor growth in eggs and cell culture (17–20). Following the loss of α2,3-Sia recognition, H3 subtypes soon exhibited reduced affinity for the prototypical α2,6-Sia receptors, namely, short-form glycans terminating in α2,6-linked sialic acid, in favor of longer chain structures terminating in α2,6-Sia and consisting of multiple N-acetyllactosamine (LacNAc) repeats either on branched structures or as linear extensions (4, 21–23).

The consequences of changes in receptor binding properties of H3N2 viruses are particularly acute with respect to the process of choosing seasonal vaccine strains and scaling up vaccine production. Clinical isolates of H3N2 strains have become increasingly difficult to propagate in cell culture and often must be passaged in MDCK-SIAT1 cells that overexpress the human α2,6 sialyltransferase (24) to achieve reasonable virus titers. These H3N2 strains appear to rapidly acquire adaptive mutations in the HA even upon minimal passage in Madin-Darby canine kidney (MDCK) cells, and these mutations can influence both receptor binding and antigenicity (25). Furthermore, as recent H3 strains agglutinate erythrocytes very poorly, or not at all, they cannot be assessed for antigenic changes by standard HI assays (25). Such HI assays, using ferret antiserum corresponding to existing vaccine viruses, have long been a standard for determining whether circulating isolates are antigenically distinct and if updated vaccine strains are warranted. The issue of matching vaccines to currently circulating viruses is complicated further as most large-scale vaccine production still involves propagation in eggs, which can also lead to changes in binding characteristics and antigenic properties.

We have recently developed the human lung shotgun glycan microarray (HL-SGM) as a method for interrogating the receptor recognition of biological substrates (26). Previous receptor binding assays have generally been based on synthetically derived receptor analogs, the biological significance of which is unknown. The HL-SGM is comprised of N-glycans isolated from complete human lungs and provides a picture of the available glycome repertoire present in the tissue. We characterized the receptor binding profile of 10 drift viruses isolated from 2001 to 2015 and of 3 clinical isolates isolated from 2017 on the HL-SGM. We also examined the binding to a defined N-glycan array (27) which contains glycan structures reminiscent of those found to be important binders for seasonal strains in the pig lung (9). Additionally, we compared the glycomes of chicken and guinea pig erythrocytes and found many of the same N-glycan structures, especially on chicken erythrocytes, that are present on the N-glycan array. This discovery explains the matching losses in affinity on the defined N-glycan array and within the HA assay. Results of analysis of binding to the Consortium for Functional Glycomics (CFG) array aligned with the previous observations regarding loss of recognition of short α2,6 sialosides and, surprisingly, showed binding to poly-N-acetyllactosamine (PL) [-3Galβ1-4GlcNAcβ1-]_n_ chains that was independent of sialic acid. Ultimately, we see some recognition of sialylated structures on the HL-SGM, many containing PL determinants similar to those described previously by others (4, 21, 22, 28). The most striking feature, however, is the discovery of robust binding of drift viruses to phosphorylated high-mannose glycans. This recognition remains consistent across the entire panel of analyzed viruses, indicating that such interactions are unaffected by antigenic drift.

## RESULTS

### Hemagglutination properties of H3N2 viruses.

**FIG 1:**
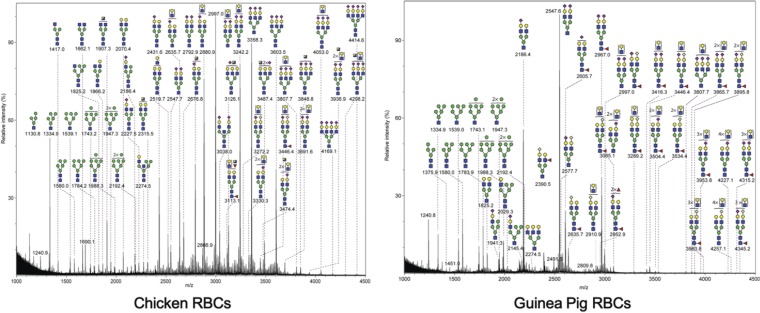
N-glycan profiles of chicken and guinea pig erythrocytes. The N-glycans on chicken or guinea pig erythrocytes were released by the use of sodium hypochlorite and analyzed by MALDI-MS. The glycan compositions were deduced based on the *m/z* value and are indicated at the top of the spectrum. Blue box, *N*-acetylglucosamine; green circle, mannose; yellow circle, galactose; purple diamond, N-acetylneuraminic acid; light blue diamond, N-glycolylneuraminic acid; red triangle, fucose.

We analyzed the receptor binding characteristics of a panel of 10 IAV H3N2 strains isolated in 2001 through 2015 and inclusive of clades 3C.1, 3C.2a, 3C.3a, and 3C.3b and a set of three clinical isolates from 2017 (Table 1). As all H3N2 isolates have exhibited drift from the original 1968 introduction of the strain, we have distinguished the subset of drift strains that we studied by analysis of the timed incorporation of RBS mutations and the corresponding effects on cell culture and HA titer. Our naming convention is outlined in Table 1 and splits our panel into two groups: a 2001-to-2002 drift group and a 2004-to-2005 drift group. Each group contains viruses that include relevant receptor binding site mutations introduced within those seasons. (Viruses in the 2001-2002 drift group include residue changes at 226 incorporated before 2001 and at 225 and 222 incorporated in the 2001-2002 season. Viruses in the 2004-2005 drift group include further residue changes at 225, 226, and also at 193 incorporated in the 2004 and 2005 seasons and carried forward.) The hemagglutination properties of the panel of drift viruses and clinical isolates are shown in Table 2 and Table 3, and our data confirm previous reports indicating that these strains no longer agglutinate chicken erythrocytes whereas some activity against guinea pig erythrocytes is maintained (14–16, 20). To examine the structures available for virus binding on the cell surfaces of these erythrocytes, glycans were isolated and analyzed by matrix-assisted laser desorption ionization–mass spectrometry (MALDI-MS) (Fig. 1). Complex and high-mannose N-glycans were present in both chicken and guinea pig erythrocytes. The guinea pig red blood cell (gpRBC) glycome contains a higher abundance of glycans with potential PL repeats as evidenced by the inclusion of only one or two sialic acids, a terminal modification, in the dominant peaks. The chicken RBCs (cRBCs) contain a greater abundance of tri- and tetra-antennary branched N-glycans, indicated by the higher prevalence of sialic acid in high-molecular-weight species. In addition, the level of core fucosylation of N-glycans is higher in the guinea pig erythrocyte glycome than in the chicken erythrocyte glycome.

**TABLE 1:** Virus strains, passage history, and strain designation

Virus name	Passage history	Nomenclature^*a*^
A/Toulouse/878/2001	MDCK-Siat1	2001-2002 drift
A/Fujian/411/2002	MDCK-Siat1	2001-2002 drift
A/Finland/486/2004	MDCK-Siat1	2004-2005 drift
A/Hong Kong/4443/2005	MDCK-Siat1	2004-2005 drift
A/Esfahan/6117/2010	MDCK-Siat1	2004-2005 drift
RG/Victoria/361/2011	MDCK-Siat1	2004-2005 drift
A/Switzerland/9715293/2013	MDCK-Siat1	2004-2005 drift
A/Finland/438/2014	MDCK-Siat1	2004-2005 drift
A/Hong Kong/4800/2014	MDCK-Siat1	2004-2005 drift
A/Bucuresti/179471/2015	MDCK-Siat1	2004-2005 drift
A/New York/55/2004	MDCK	H3N2 NY04^*b*^
A/Pennsylvania/08/2008	MDCK	H1N1 Penn08
A/Brisbane/59/2007	MDCK	H1N1 Bris07
A/Georgia/DSL1/2017	MDCK-Siat1	Clinical isolate L
A/Georgia/DSM1/2017	MDCK-Siat1	Clinical isolate M
A/Georgia/DSN1/2017	MDCK-Siat1	Clinical isolate N

a Drift distinctions based on serial incorporation of receptor binding site mutations. 2001-2002 drift, residue changes at 226, 225, and 222; 2004-2005 drift, residue changes at 225, 226, and 193.

b A/New York/55/2004 is distinct from the other drift strains at residue 193 and in its ability to grow in MDCK cells and agglutinate cRBCs.

**TABLE 2:** Hemagglutination titers of H3N2 drift strains using chicken red blood cells or guinea pig red blood cells

Virus name	Hemagglutination titer^*a*^	
	cRBCs	gpRBCs
A/Toulouse/878/2001	0	2,048
A/Fujian/411/2002	4	1,024
A/Finland/486/2004	4	2,048
A/Hong Kong/4443/2005	0	4,096
A/Esfahan/6117/2010	0	512
RG/Victoria/361/2011	0	512
A/Switzerland/9715293/2013	0	1,024
A/Finland/438/2014	0	256
A/Hong Kong/4800/2014	8	512
A/Bucuresti/179471/2015	0	1,024

a cRBCs, chicken red blood cells; gpRBCs, guinea pig red blood cells.

**TABLE 3:** Residues at key HA positions and HA titers of recent clinical isolates

Clinical isolate	HA amino acid at position:						Hemagglutination titer	
	190	193	222	225	226	227	Chicken	Guinea pig
A/Georgia/DSL1/2017 (L)	D	F	R	N	I	P	No titer	16
A/Georgia/DSM1/2017 (M)	D	F	R	N	I	P	No titer	32
A/Georgia/DSN1/2017 (N)	D	F	R	N	I	P	No titer	32

### H3N2 binding to defined N-glycan microarray.

**FIG 2:**
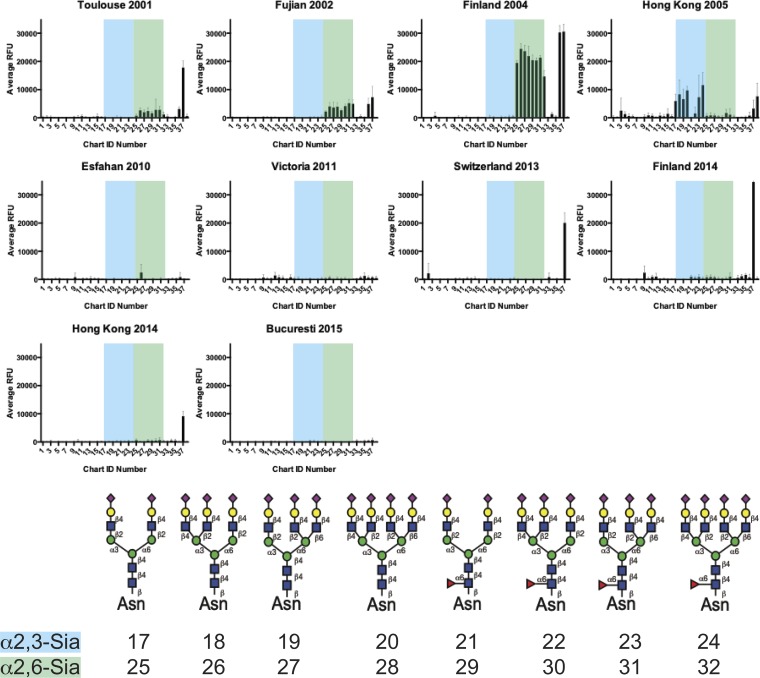
Receptor binding profile of H3N2 drift strains isolated from 2001 to 2015 on defined N-glycan array (27). The profiles for the drift strains are shown with 2,3-Sia structures highlighted in blue and 2,6-Sia structures highlighted in green. Glycans corresponding to chart IDs 17 to 32 (i.e., the Sia-terminating structures) are shown below the graphs. Error bars represent standard deviations of data corresponding to binding to replicate microarray spots. See Fig. 1 legend for cartoon key.

In order to fully characterize the binding recognition of the typical sialylated N-glycans that are considered canonical IAV receptors, we utilized a recently developed N-glycan array (27) comprised of enzymatically synthesized glycans representative of common IAV binding determinants (9). The array contains an isomeric set of bi-, tri- and tetra-antennary, nonsialylated, or fully sialylated N-glycans, with each structure terminating in either α2,3-Sia or α2,6-Sia. Additionally, each structure is present with and without a core fucose. These structures are well represented in the glycome of the chicken erythrocyte, though only the biantennary disialylated structure is abundant in the guinea pig RBC N-glycome. Previous studies on binding of the α2,6-specific wild-type (WT) X-31 virus, which contains the 1968 H3 HA, and the α2,3-specific X-31 mutant (X-31 HAM) with a 226 Leu-to-Gln substitution, illustrated the utility of this array for IAV binding (27). In general, the drift data confirm weak binding to the arrays, though the Finland 04 strain (2004-to-2005 drift) does retain strong binding to all of the α2,6-Sia-terminating glycans, regardless of the number of branches or the core fucose (Fig. 2). The α2,3-Sia binding seen with the Hong Kong 05 strain (2004-to-2005 drift) is likely due to neuraminidase, as sequence analysis of this virus reveals a residue change at position 148 (T to K) which has been described by others as a feature of NA-mediated recognition (25). Lack of virus binding to these arrays for the 2004-to-2005 drift isolates from 2010 onward suggests that the substrates for receptor binding recognition are not present (Fig. 2). This finding, combined with the loss of agglutination to chicken RBCs coated with these glycan structures, indicates that these strains no longer recognize these common sialylated N-glycan structures as receptors.

### H3N2 binding to CFG glycan microarray.

**FIG 3:**
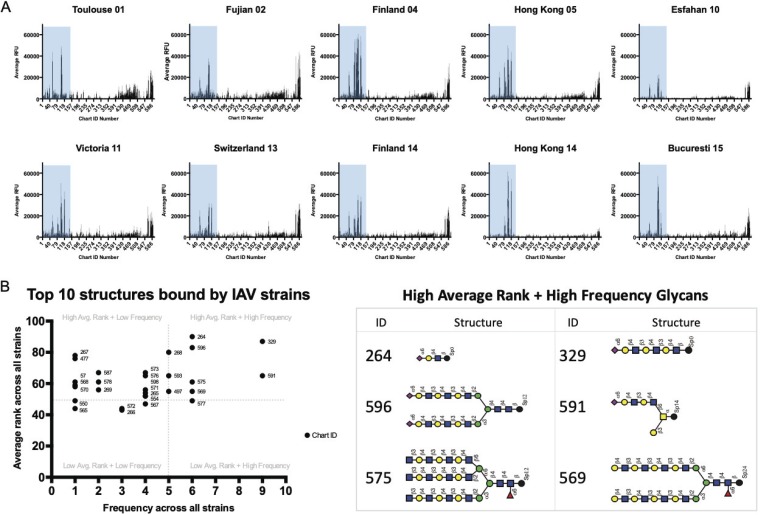
Drift viruses binding to CFG arrays. (A) Each virus was fluorescently labeled and interrogated on CFG array version 5.3. The binding profiles represent the relative intensity of the fluorescence for each glycan structure, indicating virus recognition and binding. The chart IDs have been ordered such that the sialylated glycans are grouped in chart IDs 1 to 153. (B) The top 10 structures bound by each individual virus are plotted by frequency (the number of viruses that bind each structure) and average rank (the percentage of binding for that structure for each applicable virus) and identified by chart ID. The glycan structures corresponding to the highest rank and frequency IDs are shown in the chart. See Fig. 1 legend for cartoon key.

**FIG 4:**
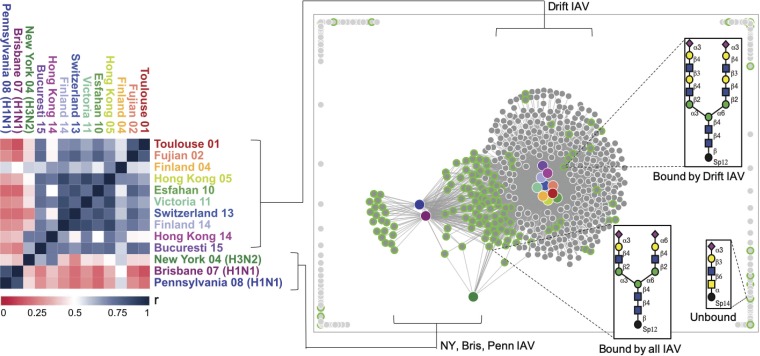
Glycan microarray comparison of drifted H3N2 strains and H3N2 NY04 and H1N1 Penn08 and Bris07 strains on CFG array. The left panel shows a correlation map encompassing binding to all structures present on the array. The right panel contains a force graph, where the color nodes indicate the virus strains and the gray nodes represent individual glycans. Binding is visualized by connections via the circles and nodes, with higher relative fluorescence units equating to shorter distance. The glycans with Neu5Ac (sialic acid) structures are highlighted in lime green. Example cartoons are included for structures bound only by the drift strains, bound by all strains, and not bound. The strain colors are consistent between the two panels.

The glycan microarray (version 5.3) from the Consortium for Functional Glycomics (CFG) is comprised of approximately 600 glycans that are chemoenzymatically generated and represent epitopes or determinants of complete glycan structures. While the N-glycans on the defined N-glycan microarray are limited to a small set of isomeric structures, notably without PL repeats, the CFG arrays include a collection of PL-containing N-glycans which are partially identified in the guinea pig glycome. Among the glycans in the collection on the CFG array, 151 contain terminal sialic acids, and both α2,3 and α2,6 linkages are well represented. Binding of the drift strains to these arrays confirmed the reduced recognition of the common biantennary disialylated substrates and revealed an increase in binding to long-chain PL glycans that has been reported previously by others (19, 21, 22, 28) (Fig. 3A). The top 10 bound structures from each drifted strain are shown in Fig. 3B, with the average rank of each bound glycan weighted against the number of drift strains (frequency) that bound to each glycan. The highest-binding glycans recognized by the most viruses are indicated in the upper right quadrant and the lowest-binding glycans among the top 10 recognized by the fewest number of viruses at the bottom left. The other quadrants represent high binders for a few viruses (top left) and low binders for many viruses (bottom right). This analysis allowed us to focus on a small subset of structural determinants that are relevant for all strains. The six glycans of the top right quadrant (high average rank and highest binding) are illustrated in the table in Fig. 3B. The dominant sialic acid linkage of the high-binding glycans is to α2,6-Sia. Interestingly, many of the determinants contained at least one lactosamine unit, some with many PL repeats, but 2 of the top 6 binding structures were not sialylated. Comparison of the drifted strains with other strains [A/Pennsylvania/08/2008 (H1N1), A/Brisbane/59/2007 (H1N1), and A/New York/55/2004 (H3N2)] showed very little correlation in glycan binding profiles, represented by the red gradient in the heat map (Fig. 4, left panel). High levels of correlation, or high degrees of similarity of binding profiles, are indicated in blue. In the right panel of Fig. 4, the binding to the CFG array is visualized via force graph. The glycans on the array are represented by the gray circles, and the viruses are represented by the larger colored nodes. Positive binding is indicated by a connection of the glycan circle with the virus node, and the distance to the node correlates with level of binding strength, with higher relative fluorescence unit (RFU) values equating to shorter connections. The sialylated glycans (circles) are highlighted in green and the nonsialylated glycans in gray and white, and the color of the virus nodes corresponds to the strain names on the correlation map. There is a distinct pattern of correspondence between the drifted strains and the H1N1 Penn08, Bris07, and H3N2 NY04 viruses, with some sialylated glycans being recognized by both sets of isolates, some recognized only by the drifted strains, and some recognized only by the Penn08, Bris07, and NY04 strains. Additionally, there are several sialylated glycans that are not bound by any of the viruses, and these populate the periphery of the graph. Representative structures of the groups (bound by drift subsets, bound by all, and not bound) are included. Strikingly, there were an abundance of nonsialylated glycans (gray circle, white outline) that were shown to be bound by the drifted strains whereas the Penn08, Bris07, and NY04 viruses recognized only sialic acid-terminating structures.

### H3N2 binding to human lung glycans.

**FIG 5:**
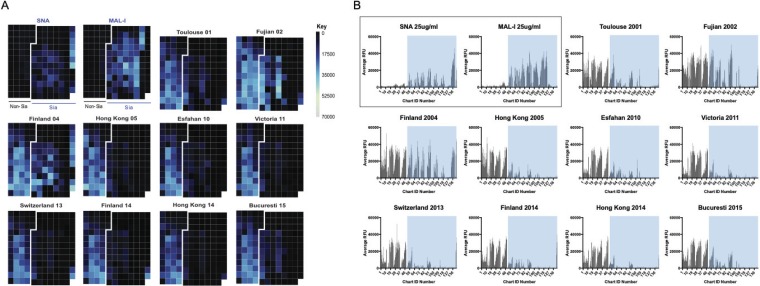
Glycan recognition of H3N2 drift strains on the HL-SGM. (A) Numbers of relative fluorescence units per glycan fraction are represented on a sliding color scale with lighter colors corresponding to greater binding intensity. (B) The receptor binding profile of each drift strain is present along with the profiles of sialic acid-recognizing lectins SNA and MAL-I. The binding of the lectins is highlighted in a blue box, which is transposed on the profiles of the viruses to indicate Sia-terminating structures. Error bars represent standard deviations of data corresponding to binding to replicate microarray spots.

**FIG 6:**
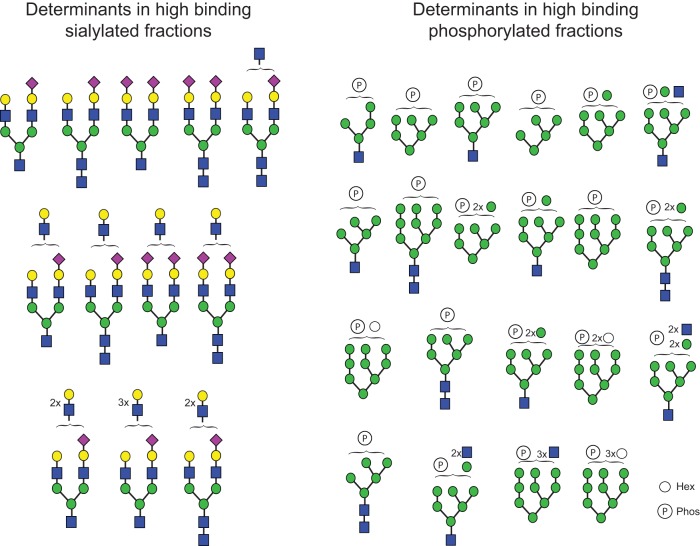
Glycans present in high-binding sialylated fractions and phosphorylated fractions on HL-SGM. For both the sialylated and phosphorylated glycan groups, the top three binding fractions shared by all drift viruses on the HL-SGM were analyzed for glycan content. Glycan structures were deduced from the corresponding *m/z* value. Some glycans appear in two or all of the top three fractions. See Fig. 1 legend for cartoon key.

Due to the nature of their design, the N-glycan array and CFG array are synthetic arrays, and results represent unknown biological significance related to the glycan structures present at the site of infection in the host. To address this limitation, we also analyzed the drift viruses on the human lung shotgun glycan microarray (HL-SGM), comprised of glycans released directly from human lung tissue (26). Interestingly, the drift strains did exhibit binding to this array, illustrated by the calendar heat map comparing average relative fluorescence unit values per glycan fraction (Fig. 5A), though they primarily recognize glycan fractions that do not overlap the sialylated structures as indicated by lectin binding profiles. This can be seen in Fig. 5B, where the blue shaded boxes represent fractions bound by SNA and MAL-1, lectins with α2,6-Sia-specific and α2,3-Sia-specific recognition, respectively (chart identifiers [IDs] 53 to 120). The glycan structures found in the fractions represented by chart IDs 1 to 52 contain high-mannose phosphorylated structures (26). Comparing the results seen for binding to the chart IDs corresponding to sialylated and control glycans (chart IDs 53 to 120 and 121 to 143, respectively), we found that a set of structures was commonly bound by all the drift strains, though some strains such as Finland 2004, Switzerland 2013, and Finland 2014 (all 2004-to-2005 drift strains) strongly bound outliers that were not recognized by the other strains, most notably the sialylneolacto-N-tetraose c (LSTc) controls [Neu5Acα2-6Galβ1-4GlcNAcβ1-3Galβ1-4Glc-2-amino-N-(2-aminoethyl)-benzamide (AEAB), chart IDs 142 and 143], which are single chain structures with one sialic acid. Among the top binding sialylated glycans, it is likely that most are biantennary with the possibility of longer chains. We cannot discount the possibility of the presence of more-complicated branched structures such as tri- or tetra-antennary monosialylated N-glycans, which are unable to be resolved by MALDI. The high-binding glycans from the phosphorylated fractions likely contain phosphorylated structures ranging from Man_5_GlcNAc_2_ to Man_9_GlcNAc_2_. Both phosphodiester and phosphomonoester bonds are possible. The glycan cartoons indicated in Fig. 6 represent the proposed structures of the glycans that make up the top three high binding fractions of both the sialylated glycan section and the phosphorylated glycan section.

### Binding of H3N2 from the 2017-to-2018 season.

**FIG 7:**
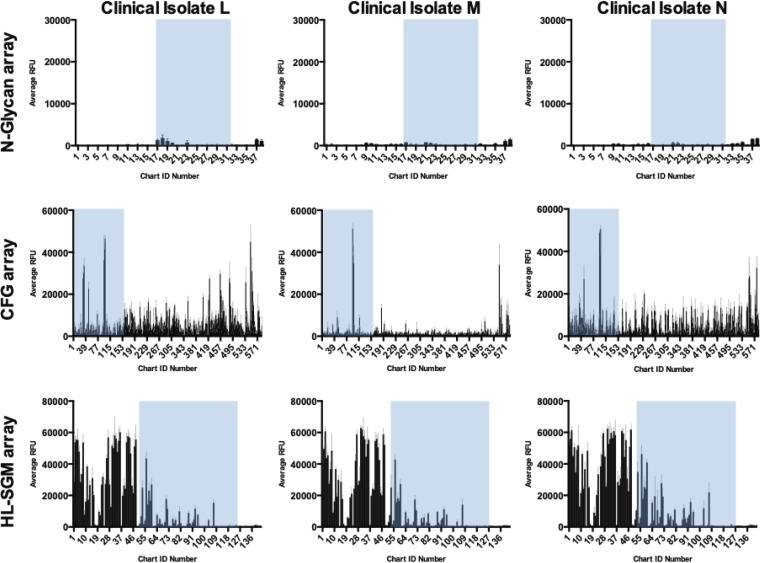
Binding of clinical isolate viruses (H3N2) collected in 2018 to glycan microarrays. (Left panel) Three strains (denoted L, M, and N) (Table 1) were tested on the N-glycan array (top row), the CFG array (middle row), and the HL-SGM (bottom row). Sialylated glycans are present in IDs 17 to 32 of the N-glycan array (See Fig. 2 for cartoons). The CFG glycans have been ordered such that sialylated glycans are listed first in chart IDs 1 to 153. Phosphorylated glycans populate chart IDs 1 to 52, and sialylated glycans correspond to chart IDs 53 to 120 on the HL-SGM. In all charts, sialylated glycans are highlighted in blue boxes. Error bars represent standard deviations of data corresponding to binding to replicate microarray spots.

We also examined three strains obtained from patients in 2017, during one of the worst influenza epidemics in several years. Sequence analysis revealed that each virus is of subtype H3N2 (Table 3). These viruses were briefly amplified in MDCK-SIAT1 cells and then used for receptor binding studies on the glycan microarrays. These viruses also did not agglutinate chicken RBCs and exhibited minimal agglutination of guinea pig RBCs (Table 3). Binding to the N-glycan array was negligible (Fig. 7), following the trend of the earlier H3N2 drift viruses. Interestingly, the profiles of these strains were highly restricted on the CFG arrays (Fig. 7) with a clear distinction between high levels of binding to a few determinants and moderate/low binding to the other available glycans. Similarly to the drift strains, these viruses were able to bind long-chain PL structures on the CFG array that did not terminate in sialic acid. Clinical isolates L and N exhibited high levels of binding to both α2,6-Sia-terminating and α2,3-Sia-terminating structures. Only the α2,6 linkage was found on the sialylated glycans in the top binders for clinical isolate M. The majority of glycan determinants recognized by this strain took the form of nonsialylated long-chain PL terminating in either a galactose residue or *N*-acetylglucosamine. On the HL-SGM (Fig. 7), the sialylated glycan recognition profiles of the three were very similar, with the same fractions appearing in the top 10 binders in slightly different orders. The three with the highest level of binding were the same for each virus and encompassed structures that were also recognized by the drift strains, including the biantennary monosialylated and biantennary disialylated structures. Each clinical isolate strain exhibited robust binding to phosphorylated glycans. Interestingly, the results representing recognition of phosphorylated structures were similar among the three isolates but, based on chart ID number were quite different from those seen with the drift strains, with only subsets of the data sets from the top binding fractions overlapping. However, many of the same predicted glycan determinants appeared in different fractions, and these more recently identified strains bound the same phosphorylated glycan structures as the drift viruses. They just bound different fractions containing those structures, indicating that other factors, including the heterogeneity of the fraction and the density of the glycans, may influence the binding. Two things to note are that the presentation of glycans on this array mimicked the abundance in the natural tissue and that the concentration of each fraction was not normalized or adjusted (26).

## DISCUSSION

Antigenic drift of H3N2 viruses circulating in the human population since 1968 has been well documented, and, with the exception of a few residues in the sialic acid binding pocket, nearly all surface residues in the globular head domain have been affected. Included among the residues that have evolved over the years are several that overlap the receptor binding domain, suggesting that some changes imposed by immune pressure could also impact the receptor binding characteristics of the virus. We have examined a panel of H3N2 viruses isolated from 2001 up to 2017, in order to track the changes in receptor recognition that accompany the antigenic drift during this period. For this purpose, we have utilized three types of glycan microarrays: the N-glycan array, consisting of α2,3 and α2,6 isomeric structures based on the general backbone of the biantennary, disialylated N-glycan previously shown to be important for IAV recognition; the CFG array, a large and diverse microarray consisting of chemoenzymatically synthesized glycan determinants; and the HL-SGM, a natural shotgun glycan microarray comprised of N-glycans obtained from healthy human lungs (26, 27). The use of these glycan arrays, combined with the structural analysis of erythrocyte glycans, has allowed us to track the trajectory of these viruses as they steadily lose recognition of conventional sialylated structures (defined as biantennary, disialylated N-glycans without repeating lactosamine units); increase recognition of long-chain PL determinants, sometimes independently of terminating sialic acid; and maintain recognition of nonsialylated phosphorylated high-mannose structures.

We examined two “sets” of viruses for this work, the first consisting of a panel of drifted strains ranging in time of isolation from 2001 to 2015 and the second of three clinical isolates obtained from patients in 2017 from the southeastern United States. The panel of drift strains was characterized in biolayer interferometry studies using sialyllactosamine as a substrate by Lin et al. (14), who observed the waning affinity for avian type α2,3-Sia receptors and subsequently for human type α2,6-Sia receptors. We confirm and extend their observations to show that the decline in sialylated glycan recognition is not limited to smaller sugars, as full N-glycan structures found on our N-glycan array are also not bound. From this representative panel of viruses, it is apparent that nearly complete loss of sialic acid recognition occurred between 2004 and 2010. The Finland 04 strain exhibited high levels of binding to α2,6 sialylated structures on the N-glycan array and strong binding to both the sialylated fractions and the phosphorylated fractions on the HL-SGM, as we observed previously with seasonal H1N1 strains (26). Finland 04 virus also exhibited robust binding to sialylated structures on the CFG array that lacked multiple lactosamine repeats, unlike the other drift viruses. These characteristics resemble those of H3N2 virus from prior years, although their reduced capacity to agglutinate chicken red blood cells provides an indication that the binding phenotype was evolving. Analysis of the Hong Kong 05 strain performed with guinea pig erythrocytes showed that the level of agglutination was higher than reported previously (14), and we also observed that this strain recognized 2,3-linked glycans on the defined array. Both results are likely due to (T/K) sequence heterogeneity at position 148 of NA in our stock virus that allows for NA-mediated agglutination (25). By the time that the 2010 strains emerged, the results from all assays showed a substantial reduction of sialylated N-glycan recognition. Our examination of a series of contemporary H3N2 strains isolated from patients during the 2017 flu season demonstrate that recent strains have maintained the binding properties of earlier strains, as they did not agglutinate chicken erythrocytes, had very low activity with guinea pig RBCs, and displayed no detectable binding to the N-glycan array. The CFG array results suggest again an ability to recognize long PL chains, though not exclusively sialylated ones. High binders were found to terminate in a variety of sugars, including sialic acid of mostly α2,6 but some α2,3 linkages, galactose, and *N*-acetylglucosamine, suggesting that the PL chain is able to interact with the virus in a nonspecific fashion. Avidity results and modeling studies suggest that the longer branched PL structures may be preferred as they are less impacted by the steric hindrance imposed by increased addition of N-glycans following mutation of the HA (4, 28).

A selection of drifted HA residues have been implicated in the progressive loss of binding to classical sialylated receptors based on biolayer interferometry avidity assays and structural studies using the receptor analog sialyllactosamine (14). Binding avidity for this substrate was substantially lower for 2004 HA than for 1968 HA and was reduced even further with mutations that arose in 2005. X-ray crystal structures of 1968, 2004, and 2005 HA:receptor complexes show that the W222R and G225D changes that occurred between 2001 and 2004, as well as the E190D mutation that emerged in the mid-1990s, were responsible for altered interactions with receptor. Comparison of the unbound 1968 structure with its receptor-bound counterpart reveals no significant conformational differences between the two, suggesting that this HA is optimally positioned to accommodate its ligand. In the 2004 HA, which has reduced affinity for sialyllactosamine compared to the 1968 HA, changes in the structure of the 220-loop are observed; W222R and G225D substitutions result in a salt bridge between the two side chains that relocates 225D closer to the receptor, where it forms a hydrogen bond to the 3-OH of Gal-2. This mutation positions the peptide carbonyl of 225D closer to the receptor, where it forms an H-bond with the 4-OH of Gal-2. The energetic cost of relocating the 220 loop to accommodate the receptor presumably results in the observed reduction of binding affinity (14). The glutamic acid side chain of residue 190 in 1968 HA also provides an additional H-bond with the 9-OH of sialic acid that is not present in the drift strains containing aspartic acid at position 190. Between 2004 and 2005, a D225N mutation arose and led to the further reduction in avidity for the receptor. The electron density for the receptor is poorly defined in the 2005 HA:receptor complex, and only the sialic acid portion can be modeled. In this structure, the sialic acid sits higher in the binding site, with fewer direct H-bonds with HA residues observed. Other changes in the receptor binding region that occurred during this drift period include S193F, L226I, and S227P, and though we have no data to directly associate these changes with changes in binding phenotype, these as well as 190D, 222R, and 225N remain present in currently circulating H3N2 strains (Table 3). At present, we do not know the biological significance of PL recognition; however, it may be possible that forming an extensive network of hydrogen bond contacts with the repeated lactosamine units of the PL chain of the glycan receptor reduces the importance of the terminal sialic acid recognition.

All of the viruses tested bound to the high-mannose phosphorylated glycans on the HL-SGM, regardless of year of isolation and state of antigenic drift/sialic acid recognition. Our previous work indicated that this phosphorylated glycan recognition is common to H1N1 seasonal and pandemic strains as well and that, for the representative A/Pennsylvania/08/2008 virus, the binding to phosphorylated glycans was likely mediated by a site separate from the HA receptor binding site (26). It is entirely possible that the recognition of phosphorylated N-glycans is a feature common to all influenza viruses and, if biologically relevant, provides a role that is functionally distinct from binding to sialylated glycans. Alternatively, binding to phosphorylated structures and/or structures such as nonsialylated PL, could aid in attachment of viruses to host cells even as human immune responses put evolutionary pressure on recognition of canonical receptors.

## MATERIALS AND METHODS

### Viruses and cells.

Virus stocks representing H3N2 drifted strains were provided by John McCauley and Yi Pu Lin of the Francis Crick Institute in the United Kingdom and Thomas Adamkiewicz and colleagues, Perimeter Pediatric Clinic, Atlanta, GA. The virus stocks were grown in Madin-Darby canine kidney cells (MDCK) cells or MDCK-Siat 1 cells that were maintained using Dulbecco’s modified Eagle’s medium (DMEM) supplemented with 5% fetal bovine serum (FBS) and penicillin-streptomycin. Briefly, 85% to 90% confluent MDCK cells grown in T175 flasks were washed twice with phosphate-buffered saline (PBS), overlaid with 10 ml of serum-free DMEM supplemented with 1 μg/ml tosylsulfonyl phenylalanyl chloromethyl ketone (TPCK) trypsin, and infected with a multiplicity of infection (MOI) of 0.01. Cells were incubated with rocking for 1 h at ambient temperature, the inoculum was removed, and 30 ml of serum-free DMEM supplemented with 1 μg/ml TPCK trypsin was added. The flasks were incubated at 37°C for 2 to 3 days until monolayers were 80% to 90% destroyed. Infected cell supernatants were clarified by low-speed centrifugation and then pelleted through a 25% sucrose cushion in NTE buffer (100 mM NaCl, 10 mM Tris, 1 mM EDTA). Purified viruses were pelleted by centrifugation using Ultra-Clear centrifuge tubes (Beckman Coulter, Indianapolis IN) and a Beckman Coulter SW32 Ti rotor at 133,548.5 × *g* for 2 h and were resuspended in 2 ml of PBS buffer at 4°C overnight. The resuspended pellets were combined and subjected to an additional spin in a SW41 Ti rotor at 55,408 × *g* for 1 h. These pellets were resuspended in 2 ml PBS, aliquoted, and frozen at −80°C. Frozen, purified virus strains were later thawed, and the levels of hemagglutination units (HAU) and titers (in plaque-forming units per milliliter) were determined using standard techniques.

### Agglutination of erythrocytes.

Chicken and guinea pig erythrocytes were acquired from Lampire Biologicals Laboratories (Pipersville, PA) or Rockland Immunochemicals, Inc. (Limerick, PA), as whole-blood preparations. Whole-blood preparations were washed two times with 1× PBS and diluted to 0.5% for hemagglutination experiments, which were performed using standard techniques. Briefly, 0.5% erythrocyte preparations were added to 2-fold serial dilutions of 50 μl of virus stock for 1 h to determine the HA titer. This procedure was done before and after labeling the virus with Alexa Fluor 488 to monitor the HA titer during the labeling process. Infectious titers of virus stocks were also determined by plaque assay on MDCK-Siat 1 cell monolayers.

### Fluorescent labeling of IAV.

Binding of fluorescently labeled IAV was performed as previously described (10, 29). Briefly, 200 μl of virus was incubated with 25 μg of Alexa Fluor 488 (Invitrogen)–1 M NaHCO_3_ (pH 9.0) for 1 h at room temperature. Labeled viruses were dialyzed against PBS using a 7,000-molecular-weight-cutoff Slide-A-Lyzer mini-dialysis unit (Thermo Scientific) overnight at 4°C. In all cases, labeled viruses were used in experiments the following day.

### Isolation of glycans from chicken and guinea pig erythrocytes.

Packed erythrocytes (200 μl) of chicken and guinea pig (Lampire Biologicals Laboratories) were resuspended in PBS buffer (pH 7.4) and washed twice. The cell pellets were collected by centrifugation (2,000 × *g*) for 10 min and lysed in water with protease inhibitor (cOmplete; Roche) for 15 min. The cell debris was recovered by centrifugation at 12,000 × *g* for 10 min and lysed again. The precipitate was taken up by PBS buffer (pH 7.4), and an aliquot was taken for bicinchoninic acid (BCA) assay to quantify the concentration of total protein. The remaining cell pellets were lyophilized and subjected to sodium hypochlorite treatment. To the cell debris of chicken and guinea pig erythrocytes, a mixture of 760 μl H_2_O and 1,140 μl 5% sodium hypochlorite and a mixture of 912 μl H_2_O and 1,368 μl 5% sodium hypochlorite were added, respectively. The mixture was incubated at room temperature (25°C) for 10 min, and the reaction was terminated by addition of 20 μl of formic acid. The products were passed through a C_18_ solid-phase-extraction (SPE) column (Waters) and washed with H_2_O. The flowthrough and H_2_O eluent were combined and applied to a Carbograph SPE column (Sigma). The column was washed with H_2_O, and the N-glycans were eluted by the use of 50% acetonitrile–0.1% trifluoroacetic acid (TFA). The N-glycan fractions were combined and dried by the use of a SpeedVac followed by lyophilization. The purified N-glycans were then subjected to permethylation and MALDI-MS analysis as described previously (30).

### Mass spectrometric analysis.

All mass spectrometric analyses of permethylated glycans from erythrocytes and AEAB-glycans from HL-SGM fractions were carried out using a Bruker Daltonics Ultraflex-II matrix-assisted laser desorption ionization–tandem time of flight (MALDI-TOF/TOF) system using reflectron mode. 2,5-Dihydroxybenzoic acid (10 mg/ml)–50% methanol was used as the matrix.

### Microarray use and analysis.

Consortium for Functional Glycomics (CFG) glycan microarrays (version 5.3), HL-SGM, and defined N-glycan arrays were used for virus binding experiments. Before assay, arrays were rehydrated for 5 min in TSM buffer (20 mM Tris-HCl, 150 mM sodium chloride [NaCl], 0.2 mM calcium chloride [CaCl_2_], and 0.2 mM magnesium chloride [MgCl_2_]). The binding experiments were carried out for viruses as previously described (9, 10, 29) for 1 h at 4°C to prevent neuraminidase activity. Biotinylated lectins (Vector Labs) were used as controls in the binding assay, and the bound lectins were detected by a secondary incubation with cyanine 5-streptavidin (Invitrogen) (5 μg/ml). For multiple arrays printed on a single slide, as used for both the N-glycan array and the HL-SGM, a ProPlate multiwell chamber (Grace Bio) was used to isolate each printed array during the assay. All arrays were scanned using a GenePix 4300A microarray scanner (Molecular Devices) equipped with 4 lasers covering an excitation (ex) range of 488 nm to 635 nm. Acquired images were quantified using GenePix Pro Microarray Analysis software and then further processed using Microsoft Excel spreadsheets as previously described (9, 10, 29). For cyanine 5 fluorescence, wavelengths of 649 nm (excitation [ex]) and 670 nm (emission [em]) were used. For Alexa Fluor 488 fluorescence, 495 nm (ex) and 519 nm (em) were used.

### GLAD data analysis.

Glycan microarray data were analyzed using the GLAD Web application to generate the heat map, correlation coefficients, and the force graphs (31). GLAD enables visual comparisons of microarray data, and the structure for the glycans can be viewed alongside the data. The settings used for the force graph were as follows: Force Range Min 1000 Max 70000, Force Strength -20, Link Distance 50.

### Data availability.

Glycan microarray data and the session file for the GLAD analysis are available on the NCFG website (https://ncfg.hms.harvard.edu/files/ncfg/files/si_glad_session_drift_plus_seasonal_iav_cfg.txt). We recommend copying and pasting the contents of the linked page as presented into a .txt file to load into GLAD as a session. The web application for GLAD is https://www.glycotoolkit.com/Tools/GLAD/. Documentation for GLAD Sessions can be found at https://glycotoolkit.com/GLAD/documentation/sessions-save-and-load/. Full documentation on how to use GLAD can be found at https://glycotoolkit.com/GLAD/documentation (31). Sequences for the clinical isolates can be found at NCBI GenBank under the following accession numbers: for DSM1.HA, MN366334; for DSM1.NA, MN366335; for DSN1.HA, MN366336; for DSN1.NA, MN366337; for DSL1.NA, MN367678; for DSL1.HA, MN367679.
